# One hundred years ago … there was a jigsaw puzzle of a farmer: teaching and learning with Kasimir Malevich

**DOI:** 10.1080/17571472.2018.1485262

**Published:** 2018-06-26

**Authors:** Francesco Carelli

**Affiliations:** a Department of Family Medicine, University of Milan, Milano, Italy

Some months ago, in London Journal of Primary Care,^1^ you may have found the article ‘There was … a goat! Impressions of learning from a young visitor to a recent Marc Chagall exhibition in Milan’

It concerned a visit with my grandchild Matteo to an exhibition of Marc Chagall. At the end of the article you were invited to consider these questions

• Does this small example demonstrate that very young people can be introduced to, involved and interested in matters not usually considered suitable for them?
• Do we believe such young people are not interested in or cannot be introduced to great art?
• What, anyway, is ‘interest’?
• How can we be sure there is none?
• What signs of interest might we see in such a young person, and how can we strive to both induce it and maintain it over time?
• How to maintain memories to as to sustain interest for further discoveries?

These questions interest Grandpa greatly who is hoping to teach Matteo by stimulating his interests. Here, we try to find an answer to the last question with a second round, introduced by a tale:

One hundred years ago there was a wizard, with a little devil as helper. One day, the little devil suggested that the wizard could become a great painter. With his magic wand he put shapes and colours on the canvas, moved a red spot here, a yellow circle there; but notwithstanding his work, the final result was horrible! A disgusting bulk of colors contrasting one against the others! The angry wizard hid the colours into a black cube. Kazimir, a Russian peasant, found in his field a black square and inside it he found coloured circles, squares, rectangles, and started to put them together, studying which could be the best combination. So, he produced beautiful pictures that made him one of the most famous painters in the world.

This was the introduction I gave this particular student: my four year old grandson, during our visit to the exhibition ‘Kazimir Malevich one hundred years after the black square’ at the Gallery of Modern and Contemporary Art in Bergamo in Spring 2016.

So, the tale ‘Kazimir and the conquest of colours’ was a teaching tool.

Another teaching tool was to find opposites: Kamizir painted the country, the workers and [one of the most remarkable among his works] the isolated red house without doors and windows. This included a return to the scheme of square. But he created also the Architekton ‘Jota’, an architectural model imagined for a future city. At the same time, we must consider the ways of the past and the new possible and future developments [in life as in medicine].

And also as in medicine, we must go beyond appearances: in Malevich when we see ‘the black cross’ we should understand that it is no more than the association or grouping of five black squares and four white squares. When we look at a patient and let him or her speak, are we able to see all these different aspects, all the components, all the different co morbidities and concurrent causes realizing the uniqueness of that patient?

When we teach our students, we push, encourage and motivate them by asking how they feel about themselves in the situations they have to face; and so it is the same with Matteo, observing Malevich’s works. I asked him:

‘What colour do you feel today?’

‘Did you ever feel yourself a black square?’

‘What would make you feel like a red circle?’

When we teach our students, we can act to create role-plays, to form groups in contrast each other, each other prepared to defend an idea or a position about what they are learning or seeing.

So, for Matteo ‘to make square’ as were the Malevich’s squares, means joining forces to defend own ideas. We can create a square for defence, make a triangle or a wedge to go on the attack, or join forces making a circle.1

**Figure:**
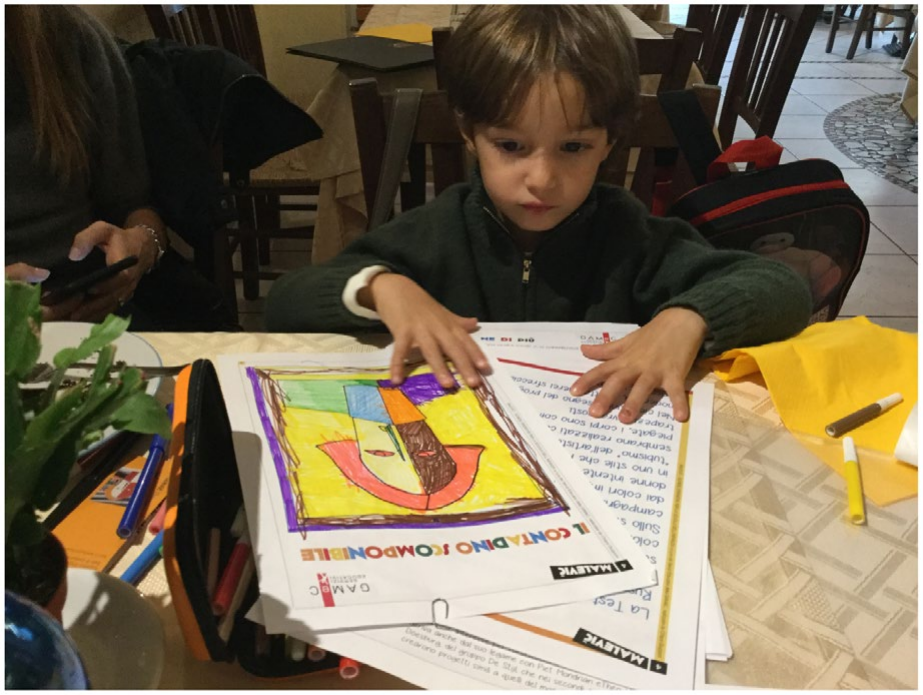


‘The head of farmer’ is the key work for Matteo also. Here, he learnt the most from the philosophy of Malevich. His artistic and philosophical school of Suprematism proclaimed that that paintings were composed of flat, abstracts areas of paint, serving up powerful and multi-layered symbols and mystical feelings of time and space. Matteo has seen the work and took a white sheet with only schematic contours of the face in where to put the colours. Here, he was able to enter colours as he wished. Then he cut the work done up and, combining different pieces like a puzzle, he was able to create new positions or new compositions with creativity.^2^

At the end, Matteo went to the great hall, where the exhibition’s curators created for the first time the atmosphere of the opera ‘Victory on the Sun’: one of the most important works in Russian Cubofuturist Theatre.

Then, he was in front of the theatrical figures drawn and designed by Malevich, and for the first time reproduced life-size in that great room. What was hypothetical, as seen in costumes drawn in pencil and seen in frames was now the reality: mannequins with the most fantastic costumes, each one different, as perhaps in medicine when the student sees for the first time real patients, each with their own large and different problems.

Matteo was really involved all the time, even listening to the audio guide tape. He asked a lot of questions.

Later, at home, he explained to his father how the artist, at the beginning painted figures, to change later into triangles, circles and squares, and then to return to the figures. But many of these figures, at that time, were painted as empty heads without eyes, mouths and noses.

He asked me the reason for the empty faces.

Would you have been able to answer him this question?

Would you be ready to find a similar explanation in observation of your student?

## Notes

1. Vol. 7, issue 3, pages 59–60.

2. Parental permission given.

