# Erratum

**DOI:** 10.1080/17571472.2018.1499425

**Published:** 2018-07-30

Carelli, F. 2018. One hundred years ago … there was a jigsaw puzzle of a farmer: teaching and learning with Kasimir Malevich. *London Journal of Primary Care,* pp. 1–2. https://doi.org/10.1080/17571472.2018.1485262

When the above article was first published online, the author was incorrectly listed as Francesco Carelli Matteo. This has now been corrected in the online version to Francesco Carelli (and Matteo).

Taylor & Francis apologizes for this error.

